# Small Molecules as Alternate Substrates for 3‐Methylglutaconylation

**DOI:** 10.1002/jmd2.70047

**Published:** 2025-10-15

**Authors:** Elizabeth A. Jennings, Irina Romenskaia, Robert O. Ryan

**Affiliations:** ^1^ Department of Biochemistry and Molecular Biology University of Nevada Reno Nevada USA

**Keywords:** 3‐methylglutaconyl‐CoA hydratase, 3MGC anhydride, HMG‐CoA lyase, metabolite acylation, primary amine

## Abstract

The leucine catabolism pathway intermediate, *trans*‐3‐methylglutaconyl (3MGC) CoA, is susceptible to a series of non‐enzymatic reactions that generate organic acid waste products and protein 3MGCylation. These reactions proceed when inborn errors of metabolism (IEM) in *HMGCL* or *AUH* lead to enzyme deficiencies. When *trans*‐3MGC‐CoA levels rise, a portion of this metabolite pool isomerizes to *cis*‐3MGC‐CoA, forming a diastereomer that is capable of spontaneous intramolecular cyclization, yielding 3MGC anhydride and free CoA. 3MGC anhydride can undergo hydrolysis to 3MGC acid or react with protein lysine residues to 3MGCylate proteins. The present study was designed to examine the ability of small molecules to react with 3MGC anhydride. An antibody directed against 3MGC was employed in vitro experiments designed to assess the ability of candidate biomolecules to attenuate the immunoblot signal intensity of 3MGCylated bovine serum albumin (BSA). When *trans*‐3MGC‐CoA was incubated in the presence of glycine, glucosamine, ethanolamine, or glutathione, each of these free amino group‐containing molecules, but not N‐acetylglucosamine or choline, induced a concentration‐dependent decrease in 3MGCylated BSA immunoblot signal intensity. It is concluded that 3MGC anhydride can react with primary amine‐containing metabolites to acylate them.

**Summary**

- IEM induced increases in 3‐methylglutaconyl‐CoA lead to non‐enzymatic acylation of small molecules.

## 1 Introduction

*trans*‐3‐Methylglutaconyl (3MGC) CoA, an intermediate in the leucine catabolism pathway, is generated from 3‐methylcrotonyl (3MC) CoA via the activity of 3MC‐CoA carboxylase (3‐MCC) [1]. Subsequently, this intermediate is hydrated by *trans*‐3MGC‐CoA hydratase (AUH; OMIM #600529), forming 3‐hydroxy, 3‐methylglutaryl (HMG) CoA. In mitochondria, HMG‐CoA is converted to acetoacetate and acetyl‐CoA by HMG‐CoA lyase (HMGCL; OMIM #246450). In non‐hepatic tissues, acetoacetate is further metabolized to yield two acetyl‐CoA. Thus, leucine is a strictly ketogenic amino acid that yields three acetyl‐CoA, which can enter the TCA cycle for energy production, providing a glucose‐sparing effect, especially in muscle [2].

**FIGURE 1:**
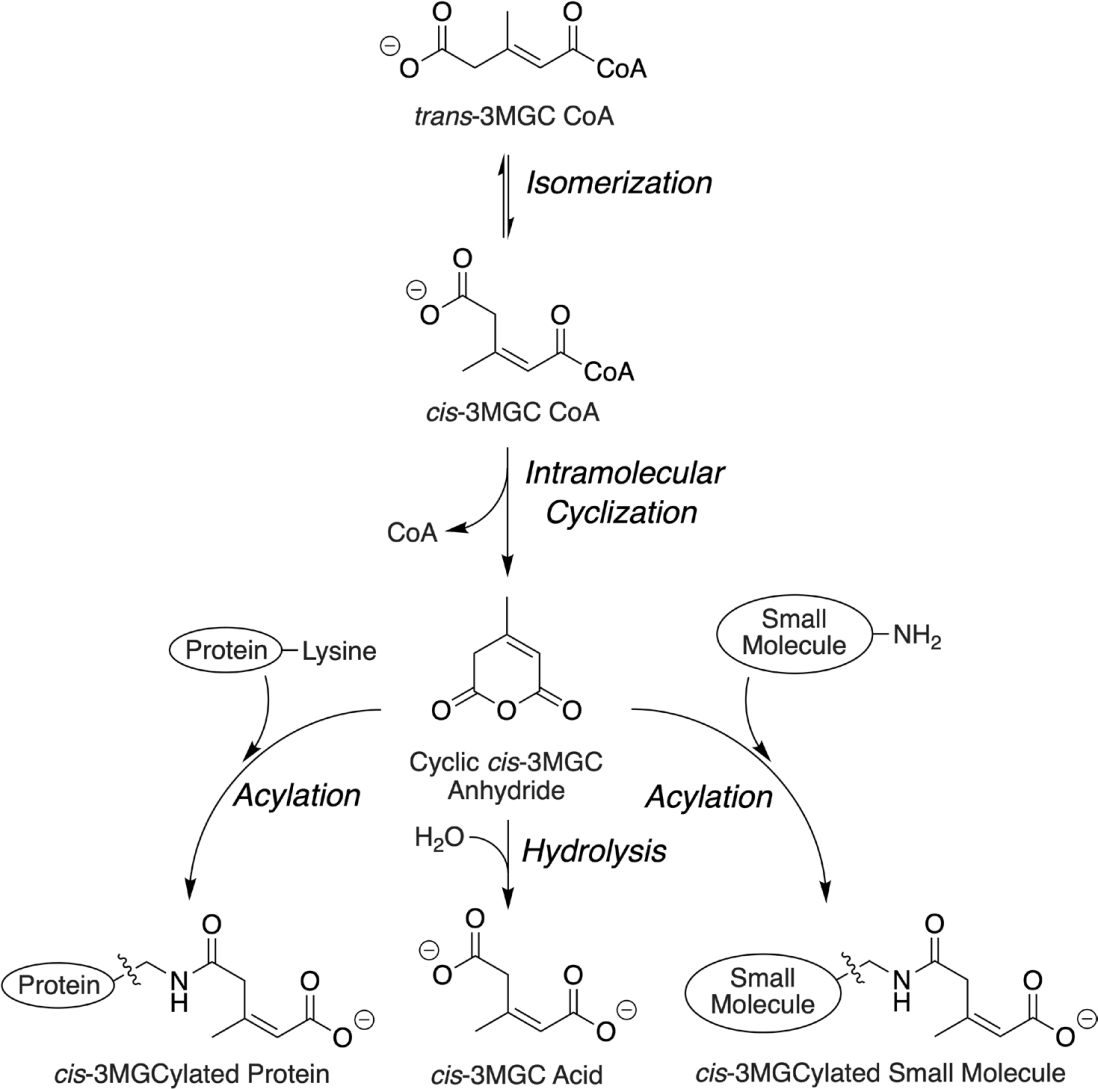
Non‐enzymatic reactions of trans‐3MGC‐CoA. The series of non‐enzymatic chemical reactions induced by the accumulation of *trans*‐3MGC‐CoA in vivo is depicted. *Trans*‐3MGC‐CoA formed as an intermediate in the leucine catabolism pathway or alternatively by the acetyl CoA diversion pathway [6] spontaneously isomerizes to *cis*‐3MGC‐CoA. A subsequent intramolecular cyclization reaction generates *cis*‐3MGC anhydride and free CoA. 3MGC anhydride has three potential fates, including hydrolysis to yield *cis*‐3MGC acid (center), acylation of protein lysine residues (left), or acylation of amino group‐containing small molecules (right).

When either AUH or HMGCL is deficient, due to inborn errors of metabolism (IEM), large quantities of 3MGC acid are excreted in urine [3]. This phenomenon is consistent with a precursor‐product relationship between *trans*‐3MGC‐CoA and 3MGC acid. Studies designed to address whether an acyl CoA thioesterase enzyme family member [4], or some other mechanism, is responsible for this conversion revealed that *trans*‐3MGC is intrinsically unstable and prone to non‐enzymatic isomerization [5]. Following isomerization to *cis*‐3MGC‐CoA, the molecule is poised to undergo intramolecular cyclization, forming *cis*‐3MGC anhydride and free CoA (Figure 1). The anhydride is susceptible to hydrolysis, which leads to the formation of the organic acid, *cis*‐3MGC acid [7]. Young et al. [8] reported that, in addition to undergoing hydrolysis, *cis*‐3MGC anhydride reacts with protein lysine residues to form a covalent adduct. Recent studies of this acylation reaction [7, 9] revealed that protein 3MGCylation is temperature and time dependent. Furthermore, studies with liver‐specific HMGCL gene disrupted mouse mitochondrial extracts showed that multiple proteins are 3MGCylated in vivo [10].

When in vitro protein 3MGCylation reactions were conducted using Tris HCl as buffer, less 3MGCylation occurred than when HEPES buffer was used [9]. Given that Tris contains a free amino group while HEPES does not, it was hypothesized that competition exists between Tris and protein lysine residues for available *cis*‐3MGC anhydride, resulting in decreased protein 3MGCylation. To assess whether physiologically relevant small molecules normally found in cells/mitochondria also function in this manner, the ability of candidate small molecules to attenuate *trans*‐3MGC‐CoA‐dependent 3MGCylation of bovine serum albumin (BSA) was assayed using α‐3MGC IgG immunoblot signal intensity as a readout. It is concluded that, once formed, cyclic 3MGC anhydride can react with primary amino groups on small molecules. These results suggest that toxic effects attributed to 3MGC acid [10] may be exacerbated by covalent modification of small molecules [11].

## 2 Materials and Methods

### 2.1 Chemicals, Reagents, and Enzymes

3‐methylcrotonyl (3MC) CoA was from Sigma Chemical Co. *Xanthamonas citri* 3‐MCC was expressed in *E. coli* and isolated as described [8]. BSA was from Millipore‐Sigma. Glycine, glucosamine, choline, ethanolamine, and glutathione were from Sigma‐Aldrich. N‐acetylglucosamine was from Thermo Scientific. 3‐MGC anhydride (4‐methyl‐2H‐pyran‐2,6(3H)‐dione) was from Biosynth Ltd. (UK).

### 2.2 3‐MCC‐Mediated Production of Trans‐3MGC‐CoA

Isolated recombinant 3‐MCC (6.7 μg per 100 μL) was incubated in buffer (50 mM HEPES, pH 8.0, 20 mM MgCl_2_, 20 mM KCl) containing 10 mM ATP, 10 mM NaHCO_3_, and 275 μM 3MC‐CoA [8]. Enzyme assays were conducted at 20°C for 2 h and stopped by removing 3‐MCC by spin filtration (10 kDa MW cutoff). The recovered filtrate, containing *trans*‐3MGC‐CoA, was then used in incubations with specified small molecules.

### 2.3 Incubation of Trans‐3MGC‐CoA With Candidate Small Molecules

Filtrate from 3‐MCC enzyme reactions (35 μL per 50 μL reaction) was incubated with various biomolecules, including glycine, N‐acetylglucosamine, glucosamine, choline, ethanolamine, or glutathione, in buffer (50 mM HEPES, pH 8.0, 20 mM MgCl_2_, 20 mM KCl). A specified range of concentrations of each biomolecule was tested. Following incubation at 37°C for 2 h, BSA was added to each reaction (0.5 mg/mL final concentration). Incubations in the presence of BSA were conducted at 37°C for 3 h or 24 h. Samples were then analyzed by α‐3MGC IgG immunoblot assay [8] to detect 3MGCylated BSA.

### 2.4 Incubation of 3MGC Anhydride With Small Molecules

Synthetic 3MGC anhydride (1 mM final concentration) was incubated with indicated concentrations of small molecules (i.e., glycine, N‐acetylglucosamine, glucosamine, choline, ethanolamine, or glutathione) in 50 mM sodium phosphate, 150 mM NaCl, pH 7.4. Samples were incubated at 37°C for 2 h prior to the addition of BSA (0.5 mg/mL final concentration). Incubations with BSA were conducted at 37°C for 3 h or 24 h, followed by α‐3MGC IgG immunoblot analysis.

### 2.5 Mass Spectrometry

Glycine (5 mM), 3MGC anhydride (5 mM) or a mixture of glycine and 3MGC anhydride (5 mM glycine, 5 mM 3MGC anhydride) were incubated at room temperature in 5 mM ammonium bicarbonate (pH = ~8) for 24 h. Each sample (1 mg/mL solution) was then subjected to direct infusion (flow rate = 5 μL/min) using electrospray ionization (ESI) with a Nanospray Flex ion source coupled to an Orbitrap Eclipse mass spectrometer (Thermo Scientific, San Jose, CA) in positive mode. The ESI spray voltage was set to 3300 V with an ion transfer tube temperature of 310°C. The MS1 was acquired within the orbitrap mass analyzer with a precursor selection range from 50–500 m/z at a resolution of 120 K. Data analysis was performed using Xcalibur software (Thermo Fisher Scientific, San Jose, CA, version 4.3.73.11).

## 3 Results

### 3.1 Effect of Glycine on 3MGCylated BSA Immunoblot Signal Intensity

**FIGURE 2:**
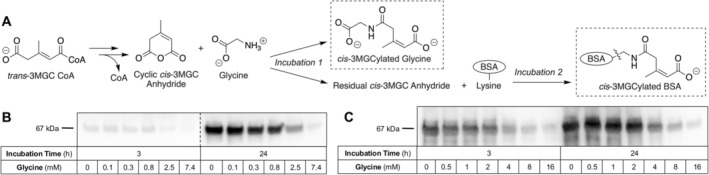
Effect of glycine on the signal intensity of 3MGCylated BSA. (A) Schematic diagram depicting the experimental design employed. “Incubation 1” refers to the incubation of 3MGC‐CoA filtrate with glycine while “Incubation 2” refers to the further incubation of this sample following the addition of BSA. Dashed box represents expected products formed. Control experiments with no glycine added were conducted in parallel. (B) Incubations were conducted as described in panel A and, following incubation, an aliquot of each sample was subjected to SDS‐PAGE and immunoblotting with α‐3MGC IgG. Dotted line indicates that the immunoblot was cut to remove extraneous lanes. (C) Incubations were conducted and processed as in panel B, except 3MGC anhydride was used in lieu of *trans*‐3MGC‐CoA filtrate.

Experiments were conducted wherein glycine was pre‐incubated with *trans*‐3MGC‐CoA‐containing filtrate followed by the addition of BSA and further incubation (Figure 2, **Panel A**). Following incubation, sample aliquots were subjected to α‐3MGC IgG immunoblot analysis [8]. The results (Figure 2, **Panel B**) show that, in both 3 and 24 h incubations, a glycine concentration‐dependent decrease in 3MGCylated BSA signal intensity was observed. The largest decrease in signal intensity was detected at glycine concentrations of 2.5 mM and above.

In separate experiments, 3MGC anhydride was used in lieu of *trans*‐3MGC‐CoA filtrate. (Figure 2, **Panel C**). In this case, a similar trend was observed, wherein glycine, at concentrations as low as 4 mM, induced attenuation of 3MGCylated BSA immunoblot signal intensity. Thus, when either *trans*‐3MGC‐CoA or 3MGC anhydride is used in reactions with BSA, glycine displayed the ability to attenuate 3MGCylated BSA immunoblot signal intensity. Mass spectrometry analysis on a similarly incubated sample containing glycine alone yielded a single mass of 76.04 Da (MH^+^). Likewise, when 3MGC anhydride was incubated alone, a single mass peak, corresponding to 3MGC anhydride (127.04 Da MH^+^), was detected. When glycine and 3MGC anhydride were incubated together, in addition to the expected peaks for glycine and 3MGC anhydride, a new peak was detected at 202.07 Da (MH^+^). This mass corresponds to that expected for 3MGCylated glycine.

### 3.2 Effect of Other Small Molecules on Trans‐3MGC‐CoA‐Dependent Acylation of BSA

**FIGURE 3:**
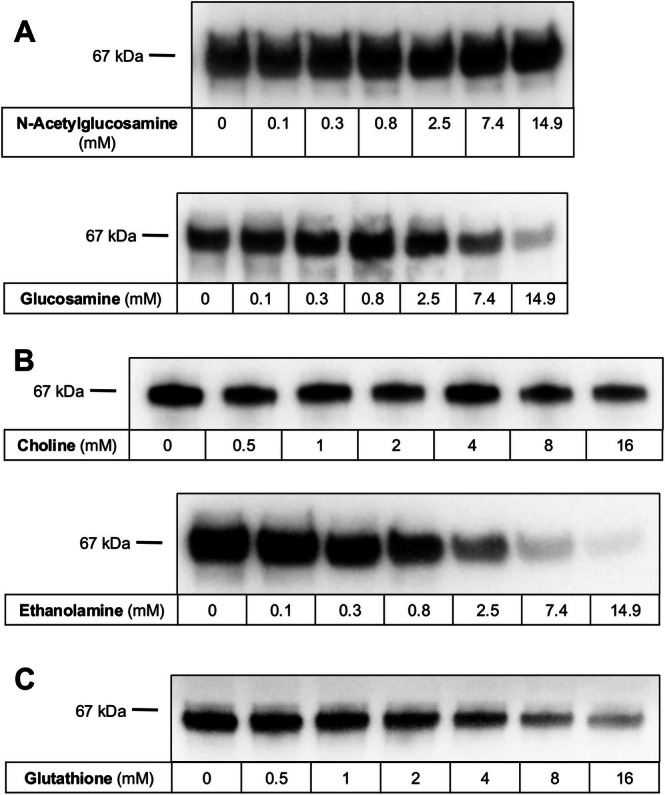
Effect of small molecules on *trans*‐3MGC‐CoA‐dependent acylation of BSA. (A) A *trans*‐3MGC‐CoA‐containing 3‐MCC reaction filtrate was incubated with indicated amounts of N‐acetylglucosamine or glucosamine for 2 h at 37°C. BSA (0.5 mg/mL) was then added, and the samples incubated for a further 24 h. An aliquot of each sample was then subjected to α‐3MGC IgG immunoblot analysis. (B) A *trans*‐3MGC‐CoA‐containing 3‐MCC reaction filtrate was incubated with specified amounts of choline or ethanolamine for 2 h at 37°C. BSA (0.5 mg/mL) was then added, and the samples incubated for a further 24 h. An aliquot of each sample was then subjected to SDS‐PAGE and immunoblotting with α‐3MGC IgG. (C) A *trans*‐3MGC‐CoA‐containing 3‐MCC reaction filtrate was incubated with specified amounts of glutathione for 2 h at 37°C. BSA (0.5 mg/mL) was then added, and the samples incubated for a further 24 h. An aliquot of each sample was then subjected to α‐3MGC IgG immunoblot analysis.

Amino sugars are abundant in cells and play important roles in glycosylated proteins and glycosaminoglycans. When increasing concentrations of N‐acetylglucosamine were pre‐incubated with *trans*‐3MGC‐CoA containing filtrate, followed by the addition of BSA and further incubation, α‐3MGC IgG immunoblot analysis revealed no effect on 3MGCylated BSA signal intensity (Figure 3, **Panel A**). By contrast, when glucosamine was pre‐incubated with *trans*‐3MGC‐CoA, in lieu of N‐acetylglucosamine, attenuation of 3MGCylated BSA signal intensity was detected at 2.5 mM glucosamine and above. These experiments, and those described below, were also conducted using 3MGC anhydride. In each case, experiments with 3MGC anhydride yielded results similar to those obtained with *trans*‐3MGC‐CoA. For clarity of presentation, only experimental data obtained with *trans*‐3MGC‐CoA filtrate are presented.

Ethanolamine is another primary amine‐containing small molecule that serves as a component of the membrane phospholipid, phosphatidylethanolamine. As a control, the trimethylated homolog of ethanolamine, choline, was also studied. Increasing concentrations of choline were pre‐incubated with a fixed amount of *trans*‐3MGC‐CoA‐containing filtrate, followed by the addition of BSA and further incubation. Immunoblot analysis of these samples revealed that choline had no effect on 3MGCylated BSA signal intensity (Figure 3, **panel B**). By contrast, when ethanolamine was examined, 3MGCylated BSA immunoblot signal attenuation was observed at 2.5 mM and above, as compared to a control incubation with no ethanolamine added.

Reduced glutathione is a tripeptide (Glu‐Cys‐Gly) in which glutamic acid is attached to the N‐terminus of Cys via its side chain carboxyl group. The concentration of glutathione in mitochondria is 10–15 mM, where it functions as an antioxidant and regulator of pathways involved in cellular homeostasis [12]. To examine the ability of glutathione to be acylated by *trans*‐3MGC‐CoA, increasing amounts of this tripeptide were incubated with a fixed amount of *trans*‐3MGC‐CoA filtrate, followed by incubation with BSA. Subsequent α‐3MGC IgG immunoblot analysis (Figure 3, **Panel C**) revealed glutathione‐dependent attenuation of 3MGCylated BSA immunoblot signal intensity at concentrations of 4 mM and above.

## 4 Discussion

In the present study, a novel assay has been employed to investigate the ability of small molecules to react with a specific byproduct of the leucine catabolism pathway intermediate, *trans*‐3MGC‐CoA. Studies have shown that *trans*‐3MGC‐CoA accumulates when IEMs lead to deficiencies in specific pathway enzymes (i.e., HMGCL or AUH). Because *trans*‐3MGC‐CoA is unstable, it is prone to non‐enzymatic isomerization to *cis*‐3MGC‐CoA. Unlike *trans*‐3MGC‐CoA, which is sterically prevented from non‐enzymatic intramolecular cyclization reactions known to occur in terminally carboxylated short chain acyl CoAs [13], *cis*‐3MGC‐CoA readily undergoes this reaction [7], yielding 3MGC anhydride and free CoA as products. The cyclic anhydride has at least two fates (see Figure 1) including (1) non‐enzymatic hydrolysis to form the organic acid 3MGC acid. Indeed, subjects with IEMs in HMGCL or AUH excrete massive quantities of this organic acid in urine [14, 15]. The second fate involves the reaction of 3MGC anhydride with protein lysine residues to covalently 3MGCylate proteins. Evidence obtained using HMGCL gene ablated mice revealed that this reaction occurs in vivo [10]. Detection of 3MGCylated proteins in this case was revealed by immunoblot analysis using a rabbit polyclonal IgG directed against a 3MGC hapten [8]. In vitro studies have shown that the incubation of *trans*‐3MGC‐CoA with BSA results in time‐ and temperature‐dependent covalent 3MGCylation of BSA [7]. More recently, the concept that non‐proteinaceous small molecules that possess a primary amine functional group may also be susceptible to acylation was proposed [10].

An assay to test this hypothesis was developed wherein the ability of various small molecules to attenuate α‐3MGC immunoblot signal intensity in incubations containing BSA and *trans*‐3MGC‐CoA (or 3MGC anhydride), was examined. When *trans*‐3MGC‐CoA is incubated with BSA for a specified time, immunoblot analysis yields a reproducible signal intensity [7]. When *trans*‐3MGC‐CoA is omitted from such incubations, however, no signal is observed. When a *trans*‐3MGC‐CoA‐containing filtrate was pre‐incubated with certain small molecules prior to the addition of BSA and further incubation, 3MGCylated BSA signal intensity was attenuated. For example, when the amino acid glycine was pre‐incubated with *trans*‐3MGC‐CoA (or 3MGC anhydride) at concentrations above a specific threshold, attenuation of 3MGCylated BSA immunoblot signal intensity was observed. Given that immunoblot signal intensity from control incubations lacking glycine were unaffected, it was hypothesized that the free amino group on glycine serves as an alternate substrate for 3MGCylation. This interpretation was confirmed by mass spectrometry analysis, which revealed that incubations of glycine and 3MGC anhydride produce a covalent glycine‐3MGC adduct. Thus, as the amount of 3MGCylated glycine increases, less *trans*‐3MGC‐CoA is available to react with BSA, resulting in a decrease in immunoblot signal intensity.

In addition to glycine, this assay method was used to evaluate other known primary amine‐containing biomolecules including glucosamine, ethanolamine, and glutathione. Importantly, chemically related control metabolites, choline and N‐acetylglucosamine, which do not possess a free amino group, had no effect on 3MGCylated BSA immunoblot signal intensity. This result supports the view that the free amino group present on glycine, glucosamine, ethanolamine, and glutathione is necessary to elicit an attenuation in 3MGCylated BSA immunoblot signal intensity. It is also noteworthy that the primary amine‐containing metabolites investigated herein are physiologically relevant biomolecules that represent distinct chemical classes, yet they produce similar results. Based on these results, it is conceivable that the abundant mitochondrial phospholipid, phosphatidylethanolamine, could be a target for acylation by *trans*‐3MGC‐CoA derived molecules. In that case, membrane permeability and structural integrity could conceivably be compromised and contribute to phenotypic features associated with IEMs in AUH or HMGCL. Likewise, 3MGCylation of glutathione could lead to deleterious effects related to disrupted redox control. It is recognized, however, that more work is required to document such effects.

The results obtained also illustrate the utility of the α‐3MGC IgG immunoblot assay. When combined with facile, indirect competition experiments, a third non‐enzymatic chemical fate of *trans*‐3MGC‐CoA has been revealed. Whereas this report provides the first evidence of this potential fate, further investigation will be required to elucidate possible effects of the chemical environment on the relative reactivity of different small molecules with 3MGC anhydride. For example, a candidate small molecule's amino group pKa may be affected by neighboring functional groups, thereby affecting its reactivity toward 3MGC anhydride. Based on parallel studies conducted using commercially available synthetic cyclic 3MGC anhydride, the results reported herein support the view that the proposed non‐enzymatic chemical reaction sequence from *trans*‐3MGC‐CoA to cyclic 3MGC anhydride is necessary and sufficient for this process to occur in vivo [10].

An important question related to the present results, and yet to be investigated, is the extent to which small molecule 3MGCylation may contribute to the manifestation of phenotypic features in subjects with IEMs in HMGCL or AUH, which include one or more of hypotonia, hypoglycemia, metabolic acidosis, and ataxia. Furthermore, aside from deficiencies in HMGCL or AUH, collectively referred to as primary 3MGC acidurias, 3MGC aciduria also occurs in numerous IEMs that are unrelated to leucine metabolism. In these IEMs, referred to as secondary 3MGC acidurias, the mutant genes adversely affect mitochondrial energy metabolism [16]. A growing number of these IEMs have been reported [6] in which *trans*‐3MGC‐CoA is generated in vivo via a novel “acetyl CoA diversion pathway” that is unrelated to leucine metabolism [17]. Future studies will reveal whether 3MGCylation of small molecules occurs in cases of secondary 3MGC aciduria and the potential impact of this modification on these disorders.

## Author Contributions

**Elizabeth A. Jennings:** conducted experiments; data analysis and interpretation; edited manuscript. **Irina Romenskaia:** conducted experiments; performed data analysis and interpretation; edited manuscript. **Robert O. Ryan:** conceived study design; performed data analysis and interpretation; prepared initial manuscript draft; edited manuscript; secured funding.

## Ethics Statement

The authors have nothing to report.

## Consent

The authors have nothing to report.

## Conflicts of Interest

The authors declare no conflicts of interest.

## Data Availability

The data that support the findings of this study are available on request from the corresponding author. The data are not publicly available due to privacy or ethical restrictions.
